# Autonomic Nervous System Responses Can Reveal Visual Fatigue Induced by 3D Displays

**DOI:** 10.3390/s131013054

**Published:** 2013-09-26

**Authors:** Chi Jung Kim, Sangin Park, Myeung Ju Won, Mincheol Whang, Eui Chul Lee

**Affiliations:** 1 Department of Emotion Engineering, Graduate School, Sangmyung University, 7 Hongji-Dong, Jongno-Ku, Seoul 110-743, Korea; E-Mails: gatsgrain@nate.com (C.J.K.); ini6030@naver.com (S.P.); dnjsaudwn@naver.com (M.J.W.); 2 Department of Digital Media, Sangmyung University, 7 Hongji-Dong, Jongno-Ku, Seoul 110-743, Korea; E-Mail: whang@smu.ac.kr; 3 Department of Computer Science, Sangmyung University, 7 Hongji-Dong, Jongno-Ku, Seoul 110-743, Korea

**Keywords:** visual fatigue, autonomic nervous system, heart rate, galvanic skin response, skin temperature

## Abstract

Previous research has indicated that viewing 3D displays may induce greater visual fatigue than viewing 2D displays. Whether viewing 3D displays can evoke measureable emotional responses, however, is uncertain. In the present study, we examined autonomic nervous system responses in subjects viewing 2D or 3D displays. Autonomic responses were quantified in each subject by heart rate, galvanic skin response, and skin temperature. Viewers of both 2D and 3D displays showed strong positive correlations with heart rate, which indicated little differences between groups. In contrast, galvanic skin response and skin temperature showed weak positive correlations with average difference between viewing 2D and 3D. We suggest that galvanic skin response and skin temperature can be used to measure and compare autonomic nervous responses in subjects viewing 2D and 3D displays.

## 1. Introduction

With the development of 3D display technology, daily accessibility to 3D images has increased. Compared to the images for 2D displays, the 3D stereoscopic image can be more exciting and appealing. Whether or not viewing a 3D display has negative effects, however, is uncertain. Such negative effects include motion sickness and visual fatigue [1–6]. As the 3D display industry expands, the potential safety concerns for 3D displays need to be addressed.

Previous studies have examined the negative effects of 3D displays by measuring physiological signals in subjects viewing 3D displays. For example, Yano *et al.* used subjective evaluations to compare the visual fatigue induced by 2D and 3D high-definition television (HDTV) [7]. Nevertheless, subjective evaluations are limited because they do not account for individual differences among subjects. To address this problem, Lee *et al.* [8], measured visual fatigue using eye blink frequency, which is analyzed in real-time by infrared image processing. Using such physiologically-based measures is advantageous because they provide objectivity and quantitative evaluation. A commonly used physiological measure is heart rate variability (HRV), which is extracted from electrocardiography (ECG) [9,10]. HRV is considered a measure of autonomic nervous system (ANS) activity. Considering visual fatigue can increase ANS activity, it may be possible to quantify visual fatigue with HRV [10]. Other studies have used electroencephalograph (EEG) to examine the effects of 3D displays. Visual fatigue induced by 3D displays might result from the increased neural processing that is needed to combine binocular images [2]. Indeed, the EEG spectrum's beta power is stronger when viewing 3D images than when viewing 2D images [11]. Similarly, physiological responses to 3D images can be quantified with evoked potentials. [12]. When viewing 3D images, the p700 component of an event-related potential (ERP) is delayed compared to those evoked with 2D images [12]. In addition to ERPs, visual evoked cortical potentials (VECP), and steady-state visually evoked potentials (SSVEP) also can be used to measure visual fatigue when viewing 3D images [4,13].

Previous studies that examine physiological responses to viewing display images only compare measures recorded in pre- and post-viewing periods. Thus, the physiological responses that occured when the display was viewed were ignored. ERP, SSVEP, and VECP are ideal measures for such responses because they can be readily obtained from subjects while viewing a visual display. Furthermore, physiological responses to 3D images might result from the image itself, rather than visual fatigue. For example, 3D images are considered more dynamic and exciting than 2D images, and might induce different reactions. HRV, an indicator of ANS activity, was found to increase with excitement, and not visual fatigue [14]. ECG, galvanic skin response (GSR), and skin temperature (SKT) also are widely used for measuring emotions. Moreover, ECG, GSR and SKT can be measured at different time points while subjects view a display, and have the added benefits of requiring fewer sensors and having fewer artifacts than EEG.

Here, we used ECG, GSR and SKT to measure physiological responses in subjects watching 2D or 3D displays. We recorded these responses for at least 1 h as subjects viewed the displays, which allowed us to compare physiological responses between 2D and 3D images. By comparing these responses, we examined whether these ANS measurements can measure visual fatigue.

## 2. Experimental Section

### 2.1. Subjects

Twenty-six subjects participated (average age: 23.3 ± 3.1) in the study. All subjects received a full study description and agreed to participate. Participants received a remuneration equivalent to $ 70.48 for completing the study. Participants were included in the study if their eyesight (corrected or uncorrected) was at least 20/25. In addition, subjects were instructed to avoid caffeine, alcohol, and cigarettes in the 12 h prior to the experiment.

### 2.2. Experimental Procedure

**Figure 1.**
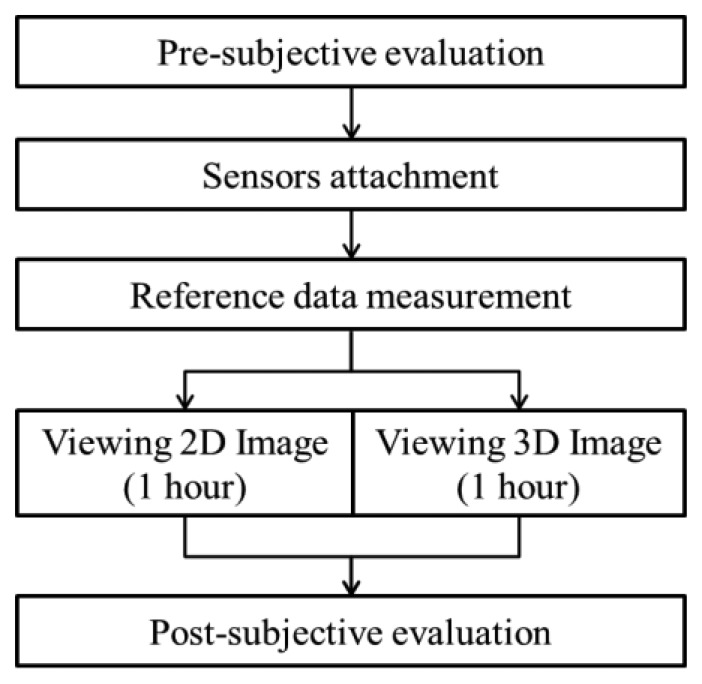
Experimental procedure.

The visual stimulus used in the experiment was a 1 h clip of a 3D enabled movie (Step-up 3D, Summit Entertainment, Touchstone Pictures in the USA, 2010). The movie was viewed with either a 2D or 3D by Blue-ray player (Samsung, Seoul, Korea) on a 40 inch LED-3DTV (Samsung, Seoul, Korea) using active shutter glasses. The 26 subjects were randomly assigned into two equal groups that viewed the movie on either a 2D or 3D display. The experimental procedure is shown in Figure 1.

**Figure 2.**
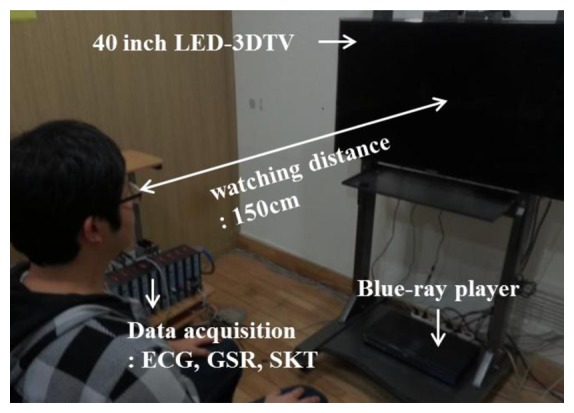
Picture of experimental setup.

As shown in Figure 2, subjects were comfortably seated in a chair, which enabled a 150 cm viewing distance. Prior to viewing the display, subjects were asked to describe their visual stress (VS), eye pain (EP), body pain (BP), and image blurring factors (IBF) using a five-point scale [15]. Participants were then fitted with ECG, GSR, and SKT sensors, and a 5 min reference data sample was recorded. Next, subjects viewed the video on either a 2D or 3D display while ECG, GSR, and SKT signals were measured. After the video concluded, subjects again described their VS, EP, BP, and IBF.

### 2.3. Data Acquisition and Processing

ECG signals were acquired with lead-sensors (Biopac, Goleta, CA, USA) placed on the left and right forearms, which also measured heart rate (HR). GSR signals were recorded with TSD203 sensors (Biopac) placed on the index and ring fingers. SKT was measured with TSD202D sensors (Biopac) placed on the thumb. All physiological data were sampled at 200 Hz using NI-DAQ-Pad9205 (National Instruments, Austin, TX, USA), and were analyzed with LabVIEW 2010 (National Instruments, Austin, TX, USA).

HR, and the average GSR and SKT were calculated for each time unit from the raw ECG, GSR and SKT signals. HR was calculated by counting the number of R-peaks occurring within a 1 min ECG signal. HR is represented as “R-R interval” which is the average interval between two adjacent R-peaks in this paper. The GSR and SKT were calculated by averaging the GSR and SKT amplitude values over 1 min periods. GSR and SKT scores were normalized by subtracting the mean values calculated from the first 1 min of data recorded in the session.

## 3. Results

### 3.1. Subjective Evaluation

**Figure 3.**
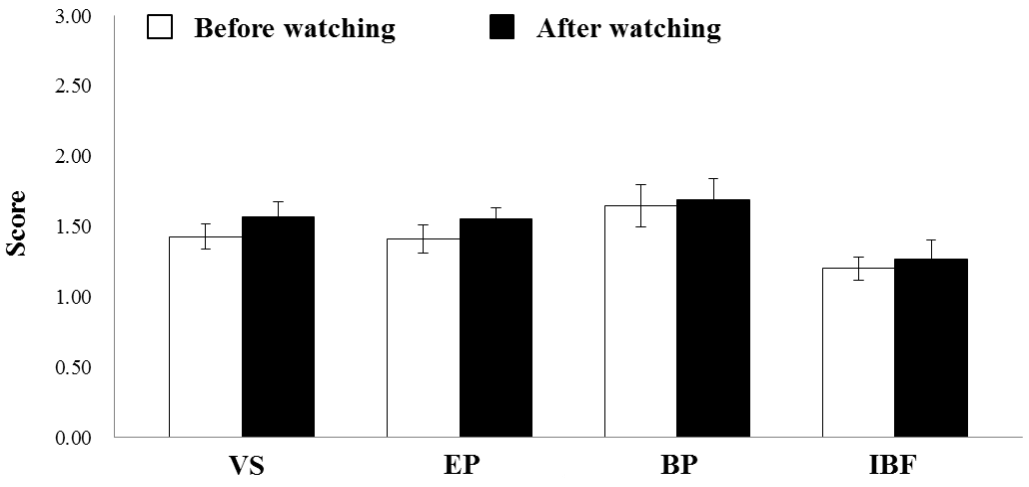
Comparison of visual fatigue scores (5-point scale) before and after viewing a 2D display. VS (Visual Stress), EP (Eye Pain), BP (Body Pain), and IBF (Image Blurring Factors).

**Figure 4.**
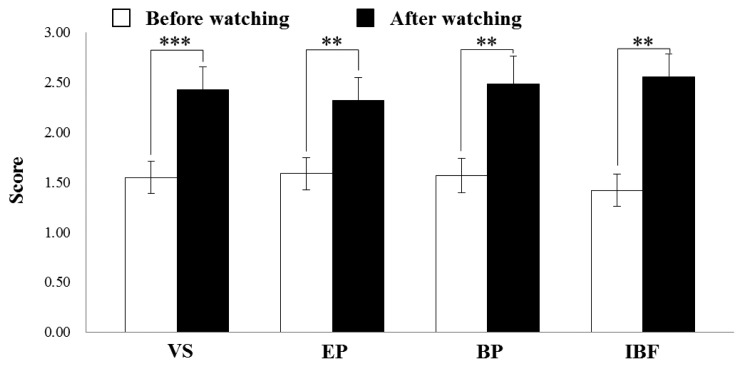
Comparison of visual fatigue scores (5-point scale) between before and after viewing a 3D display. (** and *** mean statistically significant at confidence level of 99% and 99.9%, respectively).

As shown in Figure 1, pre- and post- subjective evaluations for each subject were performed in our experiment. At result, we observed no change in the subjective evaluation after subjects viewed the 2D display (Figure 3). In contrast, we observed VS, EP, BP, and IBF all increased significantly after viewing the 3D display (Figure 4; VS: *p* = 0.001, EP: *p* = 0.005; BP: *p* = 0.004, IBP: *p* = 0.003; *t*-test). Especially, we confirmed visual fatigue phenomenon caused by viewing 3D display through the significantly increased VS compared with the other factors. Here, the *t*-test is one of the most conventionally used hypothesis tests. In general, this test is used to validate whether the average difference between two groups is significant or not. For example, *p*-value of 0.001 means that two samples is different at the confidence level of 99.9%. At result of watching 3D display, subjects reported more visual fatigue after watching display than subjects that viewed the 2D display.

### 3.2. Object Evaluation Based on Autonomic Nervous System Responses

We examined changes in R-R interval, GSR, and SKT responses during the 1 h period in which subjects viewed the 2D and 3D displays. In addition, we calculated correlations from the two dimensional feature distribution (*x_2D_t_*, *y_3D_t_*) at the *t-*th minute to quantify the relationship between these physiological measures and the display type. Considering the video stimulus was the same for the 2D and 3D groups, the correlations should be similar unless visual fatigue differed between groups.

**Figure 5.**
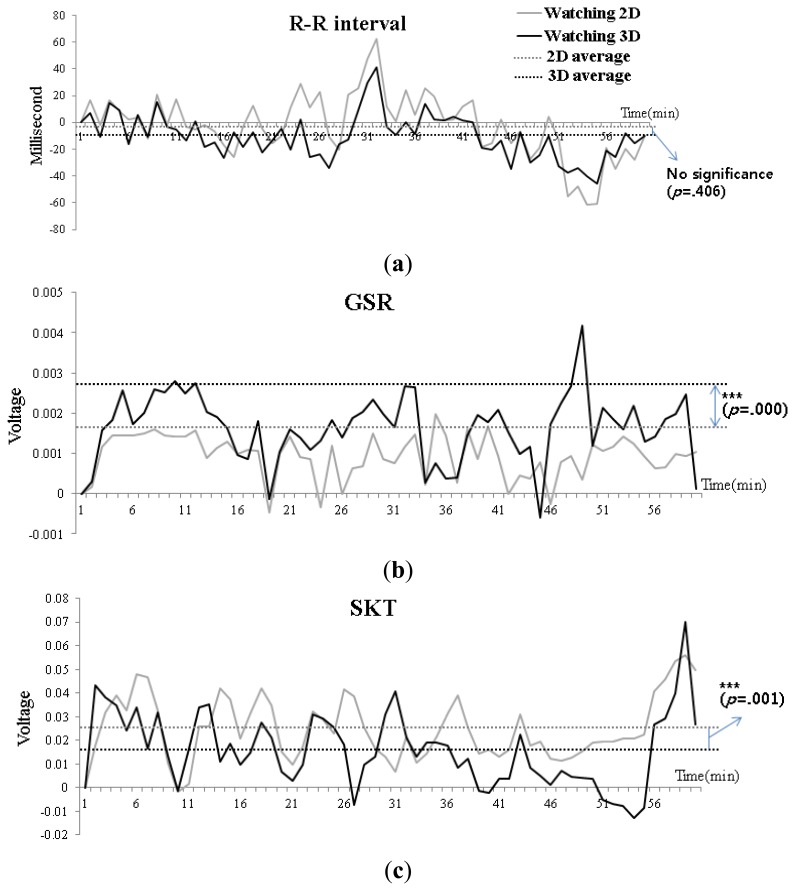
Temporal characteristics of autonomic nervous system responses in subjects viewing 2D or 3D displays (*** means statistically significant at confidence level of 99.9%). (**a**) R-R interval; (**b**) GSR; (**c**) SKT.

**Figure 6.**
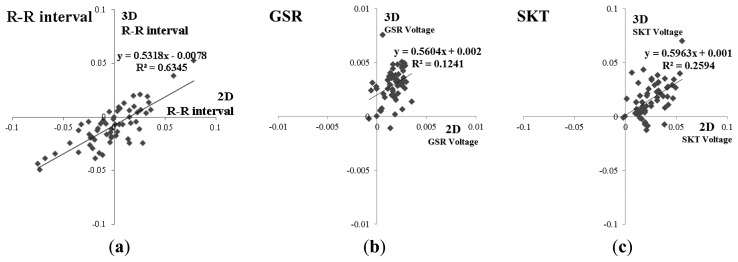
Correlational analyses of two dimensional feature distributions of 2D and 3D responses *vs.* time. (**a**) R-R interval; (**b**) GSR; (**c**) SKT.

The temporal characteristics for R-R interval showed little variation during the video presentation, and were similar between the 2D and 3D groups (Figure 5a; *p* = 0.406). This trend is evident in Figure 6a, which indicates the correlation coefficient for R-R interval between the 2D and 3D groups was 0.797, which demonstrates a strong correlation.

We observed the temporal characteristics for GSR were less similar to those observed between 3D and 2D groups for R-R interval (Figure 5b). The statistical analysis indicated that GSR differed significantly between the two groups, but this difference was small *p* = 0.000. As Figure 6b, demonstrates, the correlation coefficient for GSR between the 2D and 3D groups was 0.352, which indicates a weak correlation between these two groups.

The temporal characteristics of SKT during the display period are shown in Figure 5c for both 2D and 3D groups. The average SKT was similar between groups during the display period (*p* = 0.001). Similar to GSR, there was significant difference between 2D and 3D groups in terms of SKT. As shown in Figure 6c, the correlation coefficient for SKT between 2D and 3D groups was 0.509, which indicates a strong correlation.

## 4. Discussion and Conclusion

We examined the feasibility of ANS responses for comparatively measuring the amount of visual fatigue caused by cognitive load in individuals viewing a 2D or 3D display. Previous studies have measured cognitive fatigue in subjects viewing a 3D display [12,13], They did not, however, perform these evaluations as subjects viewed the display because the subjects performed an additional task to measure cognitive load. In the present study, we measured ANS responses, including ECG, GSR and SKT, to estimate visual fatigue while subjects viewed the 2D and 3D displays. Previous studies have examined visual fatigue by measuring ANS responses, but they did not examine responses to 3D displays [16,17].

**Figure 7.**
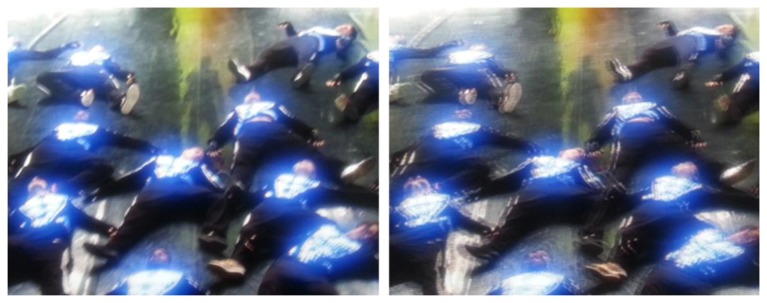
Still image examples for 2D (**left**) and 3D (**right**) displays.

**Figure 8.**
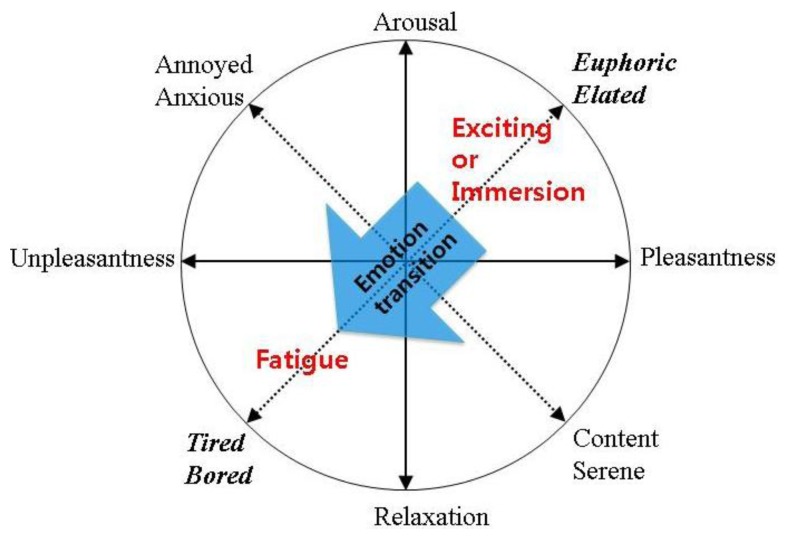
Emotion transition from immersive or exciting to fatigue.

As shown in Figure 7, we presented the same visual stimulus to both groups to eliminate confounding variables that could be introduced with different videos. This allowed us to focus on the emotion transition between exciting or immersion to fatigue at traditional emotion model [18] in Figure 8 as continuous 3D perception could induce fatigue. This is consistent with previous findings that visual fatigue could result from immersion in work [19].

We observed the temporal R-R interval characteristics were similar between 2D and 3D display groups. Heart rate appeared to be unaffected by the display type. We therefore suggest that heart rate is ineffective for comparing responses to 2D and 3D displays. In contrast, we observed that the temporal characteristics of GSR and SKT differed between subjects viewing 2D and 3D displays. We therefore suggest GSR and SKT can estimate fatigue related responses, and can be used to compare responses to 2D and 3D displays.

We observed changes in ANS responses, as quantified by ECG, GSR, and SKT, while subjects viewed a 2D or 3D display. We found evidence that indicates that GSR and SKT, but not R-R interval, can be used to measure and compare responses to 2D and 3D displays. In future studies, we will further examine specific factors that might influence these responses. In addition, we will explore multimodal features that might be combined to improve the accuracy of response classifications.
